# The importance of cellular and exosomal miRNAs in mesenchymal stem cell osteoblastic differentiation

**DOI:** 10.1038/s41598-021-85306-2

**Published:** 2021-03-15

**Authors:** Sajjad Shirazi, Chun-Chieh Huang, Miya Kang, Yu Lu, Sriram Ravindran, Lyndon F. Cooper

**Affiliations:** Department of Oral Biology, College of Dentistry, University of Illinois at Chicago, 801 S Paulina St., Room 561C, Chicago, IL 60612 USA

**Keywords:** Cell biology, Molecular biology, Stem cells, Medical research

## Abstract

The differentiation of osteoblasts is under complex regulation that includes autocrine and paracrine signaling from MSCs. Exosomes are important components of the MSC secretome and their cargo contains numerous miRNAs. In this study, the importance of MSC miRNAs in modulation of osteoblastic differentiation was examined by global reduction of miRNA biosynthesis in Dicer knock down hMSCs. We additionally impaired hMSC responses to miRNAs by knockdown of Argonaute 2 expression. Knockdown of Dicer and Argonaute 2 both reduced osteoblastic differentiation of hMSCs. This was observed at the levels of hMSC culture mineralization and osteoblastic gene expression. The treatment of Dicer deficient hMSCs with wild type hMSC exosomes effectively recovered the impaired osteoblastic differentiation. Dicer knockdown reduced the quantity and diversity of miRNAs present in hMSC exosomes. miRSeq data and KEGG analysis implicated the miRNA-dependent effects on multiple osteoinductive pathways in Dicer deficient cells, including the Hippo signaling and TGF-beta signaling pathways. Treatment of hMSCs with mimics of miRNAs significantly downregulated in Dicer knockdown cells recovered functions of exosome-mediated signaling in hMSCs. These results indicate that hMSC exosomes exert miRNA-dependent control that contributes to osteoblastic differentiation.

## Introduction

Bone regeneration is dependent on the recruitment and differentiation of mesenchymal stem cells (MSCs) that perform requisite immunomodulatory and regenerative roles as they differentiate to bone matrix forming and mineralizing osteoblastic cells^1^. The regulation of MSCs is complex and is governed local and systemic factors. Locally, MSCs are influenced by paracrine and autocrine soluble signals, cell-to-cell signals, insoluble signals of the extracellular matrix, and extracellular mechanical cues. The soluble paracrine and autocrine signals are represented by components of the cell secretome. Most notable among these are osteoinductive cytokines such as the family of bone morphogenetic proteins (BMPs)^2^. While BMPs have gained prominence due to their use in regenerative medicine, many other growth factors and cytokines influence MSC function in bone regeneration^3^.

More recent investigations of paracrine function have identified extracellular vesicles known as exosomes as additional components of the secretome that are involved in cell to cell signaling. Exosomes are 30–150 nm, bilayer lipid vesicles formed by the processing of endosomal membranes to form multi-vesicular endosomes that fuse with the plasma membrane to release exosomes filled with specific cargo. The exosomal cargo is composed of proteins, mRNAs, miRNAs and other small mRNAs^4^. Many proteins are associated with exosome biosynthesis and many are common among exosomes from diverse sources. Exosomes are not known to contain cytokines or growth factors.

miRNAs are small non-coding RNAs (21–23 nucleotides) that function to regulate the expression of target genes by repression of gene transcription or by targeted degradation of specific mRNAs or by inhibiting protein translation. The miRNA cargo of MSC exosomes is representative of the parental cell status and is specific to the cell type and cellular state^5^. Individual miRNAs function in regulation of gene expression and cell physiology^6^ and have used knockout strategies, synthetic miRNA mimics or their antagomirs to identify the functions of individual miRNAs. However, there is a controversy in terms of the importance of miRNAs and their contribution to the differentiation of MSCs as well as the role of miRNAs in MSC derived exosomes.

miRNA biosynthesis is a multistep process that involves the processing and packaging of small non-coding RNAs within the exosome^7^. They are RNA polymerase II/III transcripts that are largely intragenic and reside within introns and are transcribed in concert with or independently of the surrounding gene. Pre-miRNAs are first cleaved by Drosha in concern with the RNA binding protein DGCR8. These hairpin pre-miRNAs are exported to the cytoplasm where the RNA endonuclease Dicer removes the terminal loop, resulting in a duplex miRNA. Both the 5p and 3p miRNA strands are loaded into the Argonaute proteins (AGO1–4). When loaded onto Argonaute proteins, the miRNA strands are able to direct gene regulation. This is known as the minimal RNA induced silencing complex (RISC) and binding of the miRNA strand to the target mRNA sequence (miRNA response element; MRE) with fully complementarity, induces AGO2 endonuclease activity and resultant mRNA cleavage^8^.

miRNAs are important in cell function. The inability to generate Dicer1-null embryonic stem cells suggests its function in generating miRNAs is essential to early mouse development^9^. Subsequent conditional knockout of Dicer in ES cells indicated that Dicer participates in fundamental processes including stem cell differentiation^10^. Recent reviews indicate that conditional knockout of dicer influences development of various organ systems, again implicating the role of miRNAs in fundamental control of cell function. Regarding bone physiology, Dicer1 ablation using Osterix-cre or Runx2-cre resulted in skeletal growth impairment, post-natal bone formation and reduced osteogenesis^11^. These studies implicate miRNAs in the regulation of development, including MSC function in bone formation. miRNA function in gene silencing is dependent on AGO2 function and the disruption of the Eif2c2 gene encoding AGO2 results in developmental defects^12^. The importance of AGO2 is underscored in the context of erythrogenesis, where AGO2 is solely expressed^13^. The function of AGO2 in stem cell differentiation to the osteogenic lineage is implicated by the alterations in AGO gene expression^14^. Direct investigation of AGO2 function during MSC differentiation has not been reported.

To address the possible function of miRNAs in MSC differentiation and MSC exosomal function, we have knocked down Dicer and AGO2 in MSCs and evaluated them in a series of experiments aimed at identifying the roles of MSC miRNAs in osteogenic differentiation of MSCs.

## Results

### Knock-down of Dicer and Argonaute2

**Figure 1:**
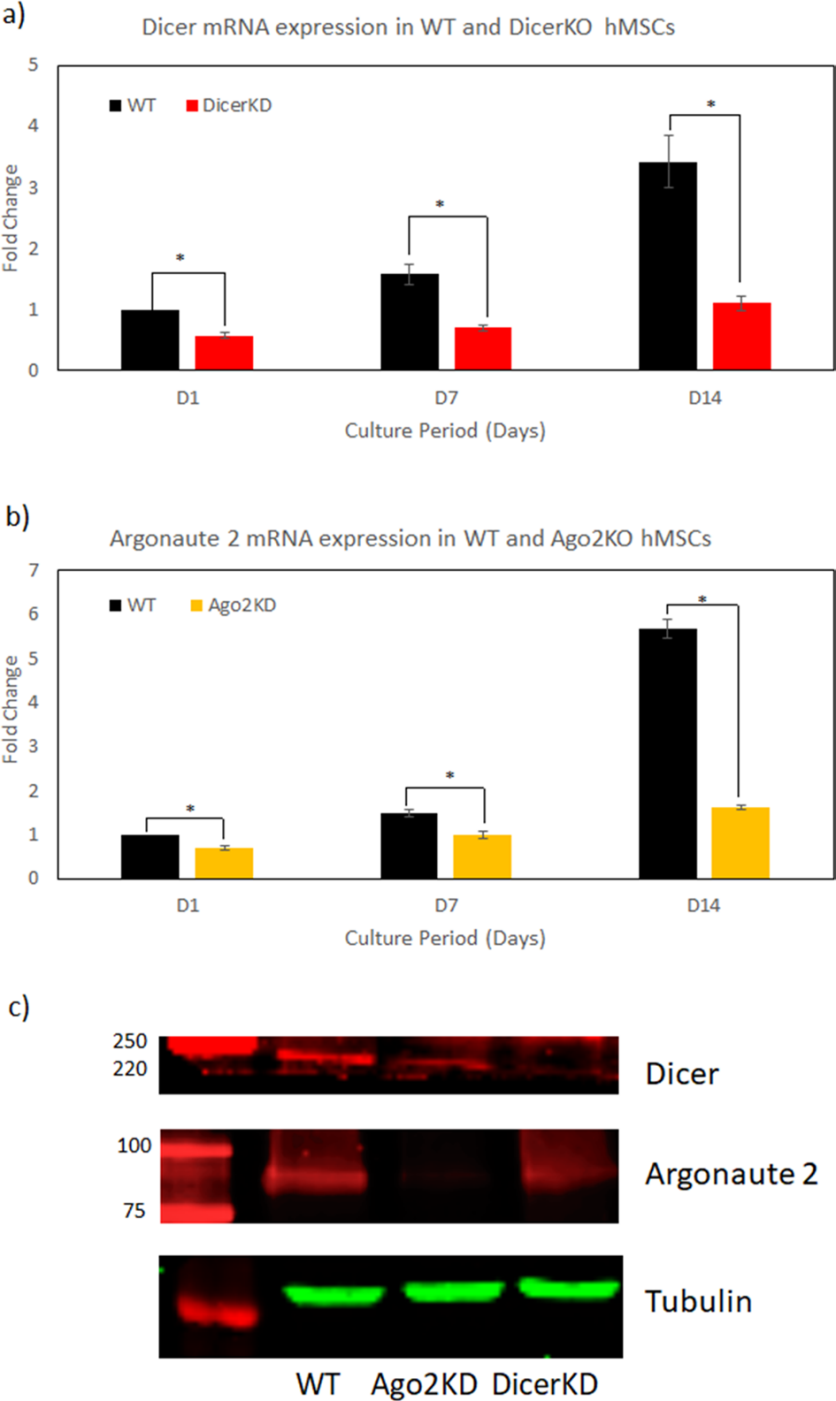
Characterization of Dicer and AGO2 knockdown. (**a**) Relative expression of Dicer mRNA was significantly downregulated (*P < 0.01) in DicerKD compared to WT hMSCs after 1, 7 and 14 days cultured in osteogenic medium. (**b**) Relative expression of Argonaute2 mRNA was significantly downregulated (*P < 0.01) in AGO2KD compared to WT hMSCs after 1, 7 and 14 days cultured in osteogenic medium. Fold changes are calculated relative to WT day 1 and Mann–Whitney U test was utilized for statistical analyses. (**c**) Immunoblotting showed successful knockdown of Dicer and Argonaute2 proteins in DicerKD and AGO2KD cells, respectively. Regions of interest of scans of one nitrocellulose blot probed with either anti-Dicer or anti-AGO2 or anti-tubulin primary antibody and corresponding secondary antibodies are shown.

Dicer and AGO2 were successfully knocked-down both in mRNA and protein levels. Figure 1a demonstrates that Dicer mRNA levels were significantly (*P* < 0.01) lower in DicerKD relative to WT hMSCs up to 14 days of culture in osteogenic medium. AGO2 mRNA levels were significantly (*P* < 0.01) lower in AGO2KD relative to WT hMSCs up to 14 days of culture in osteogenic medium (Fig. 1b). Immunoblotting demonstrated that protein levels of Dicer and AGO2 were reduced in DicerKD and AGO2KD hMSCs, respectively (Fig. 1c).

### Proliferation of DicerKD and AGO2KD hMSCs

**Figure 2:**
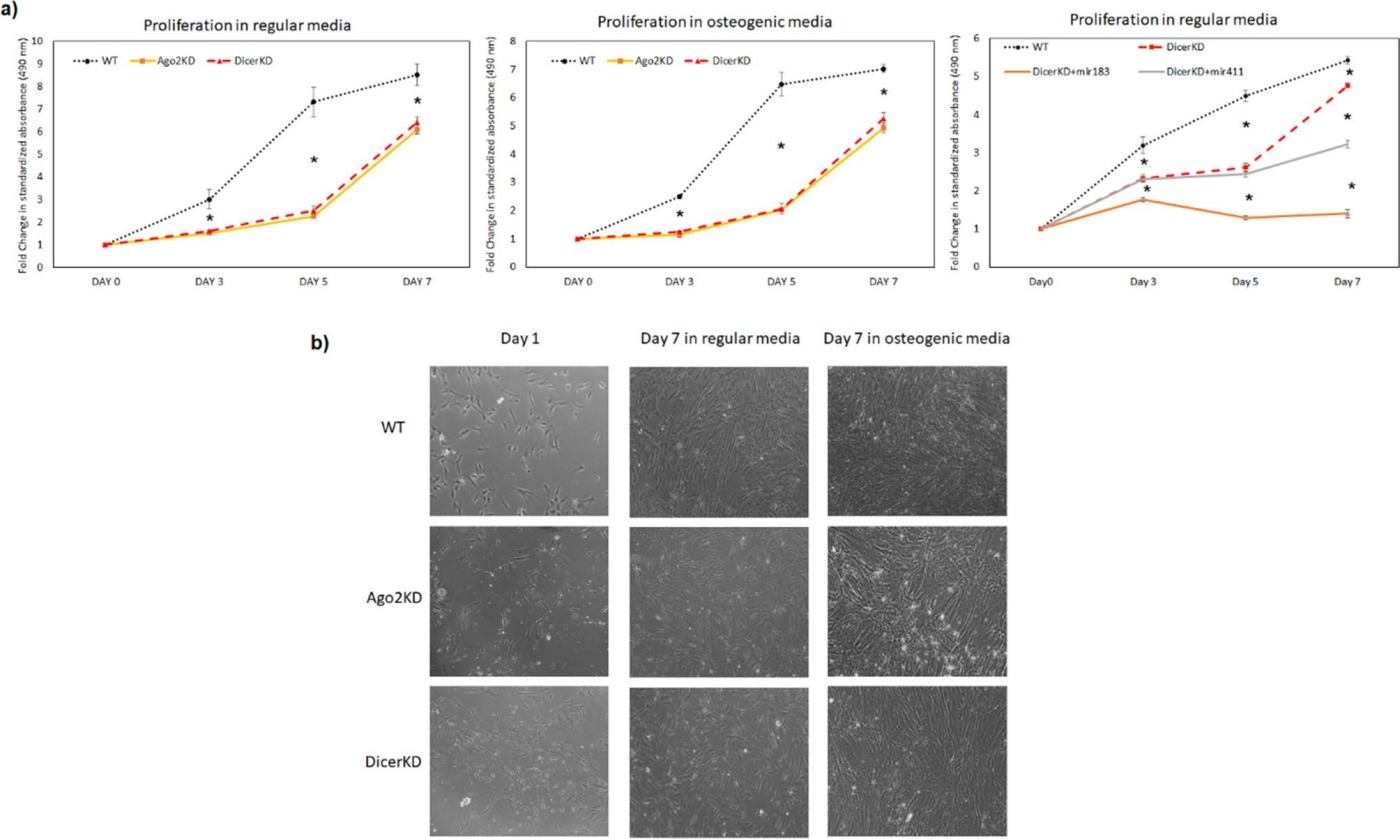
Proliferation experiments. (**a**) Proliferation of WT, DicerKD and AGO2KD hMSCs in regular growth medium and osteogenic medium was assessed using a colorimetric method. The graphs demonstrate standardized fold change in absorbance at 490 nm compared to day 1 in each cell type. (**b**) Morphology of WT, AGO2KD and DicerKD hMSCs at day 1 and 7 after growth in regular and osteogenic medium. All three cell types showed similar morphology under microscopic observation. *Denotes to statistically significant difference (*P* < 0.01) as tested with one-way ANOVA and Tukey's post-hoc test.

Measurement of proliferation of WT, DicerKD and AGO2KD hMSCs in regular growth and osteogenic medium demonstrated that WT hMSCs had greater proliferation compared to DicerKD and AGO2KD hMSCs. DicerKD and AGO2KD hMSCs had similar proliferation capacity. The miR-183-5p mimic impaired the proliferation of DicerKD hMSCs and miR-411-5p mimic significantly (*P* < 0.01) reduced the proliferation of DicerKD hMSCs (Fig. 2a). The morphology of WT, DicerKD and AGO2KD hMSCs after 1 and 7 day of growth in regular and osteogenic medium were similar at the microscopic level (Fig. 2b).

### Mineralization of DicerKD and AGO2KD hMSCs following osteoinduction

**Figure 3:**
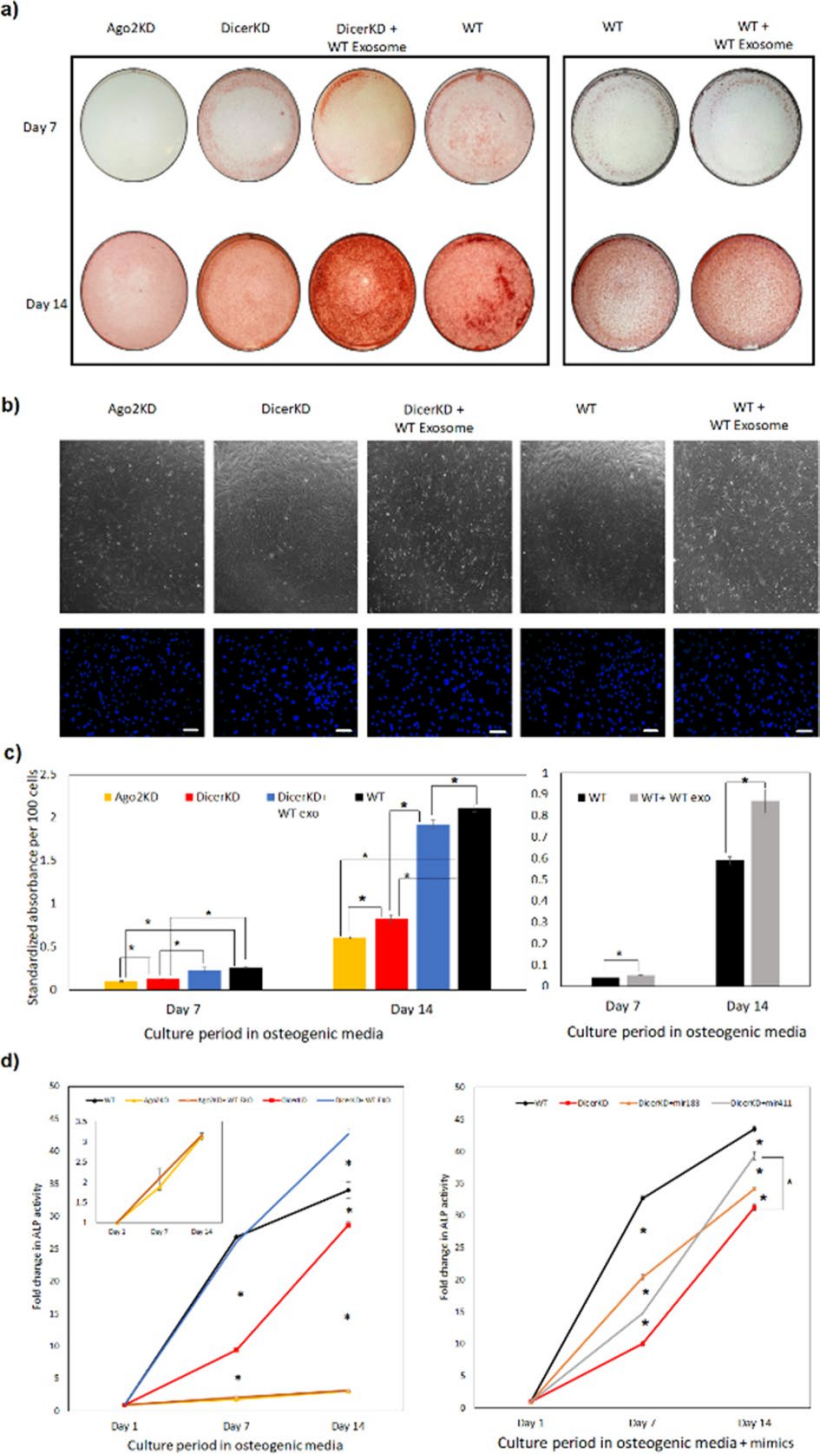
Differentiation experiments. (**a**) Alizarin Red staining demonstrate impaired calcium deposition in AGO2KD cells after 7 and 14 days of culture in osteogenic medium. DicerKD cells produced less calcium deposits compared to WT hMSCs at day 7 and 14. Complementation of DicerKD and WT hMSCs with WT exosomes considerably improved calcium deposition. (**b**) Brightfield microscopic images of experimental groups at day 7 captured at 4 × magnification demonstrated similar confluency level. DAPI staining showed similar number of nuclei per field among different groups. White scale bars are equal to 100 µm. (**c**) Quantitation of Alizarin Red staining revealed a statistically significant (*P < 0.01) difference among experimental groups. (**d**) ALP activity was measured using 45 µg of protein from each sample and calculated as standardized fold change at day 7 and 14 compared to day 1 in each group. ALP activity was significantly lower (*P < 0.01) in AGO2KD and DicerKD groups compared to WT hMSCs at day 7 and 14 in osteogenic medium. Complementation of DicerKD hMSCs with WT exosomes restored ALP activity at day 7, and increased ALP activity significantly (*P < 0.01) at day 14 compared to WT hMSCs. Complementation of AGO2KD hMSCs with WT exosomes did not significantly change ALP activity at days 7 and 14 (insert: magnified plot of ALP activity at day 7 and 14 for AGO2KD hMSCs). The addition of miR-183-5p and miR-411-5p mimics to DicerKD cells significantly (*P < 0.01) increased ALP activity at day 7 and 14. Fold changes in ALP activity was calculated compared to day 1 within each group. One-way ANOVA and Tukey's post-hoc test was used for all statistical analyses.

Mineralization was evaluated by Alizarin Red staining as illustrated by representative images of WT, DicerKD and AGO2KD hMSCs stained at day 7 and 14 of culture in osteogenic medium (Fig. 3a). Figure 3b demonstrates that the experimental groups had similar confluence level and similar cell numbers at day 7. Quantitation of Alizarin Red staining revealed the significant (*P* < 0.01) reduction in AGO2KD hMSC mineralization at day 7 and 14 compared to DicerKD and WT hMSCs. DicerKD cell layers were also significantly less mineralized (*P* < 0.01) in comparison to WT hMSCs at day 7 and 14 (Fig. 3c). To further implicate the exosome cargo in the regulation of mineralization, DicerKD hMSCs were supplemented with culture-day matched WT hMSC exosomes (supplementary Fig. 1 shows the characterization of exosomes). Complementation with WT exosomes significantly (*P* ≤ 0.01) enhanced calcium deposition of DicerKD and WT hMSCs at both day 7 and 14.

### Alkaline phosphatase (ALP) activity

ALP activity is a well-known measure of osteogenic differentiation of MSCs^15^. ALP activity was significantly higher (*P* < 0.01) in WT cells compared to DicerKD and AGO2KD hMSCs after 7 and 14 days of culture in osteogenic medium. A restoration in ALP activity was observed after complementation of DicerKD hMSCs with WT exosomes at day 7, and no significant difference was observed between WT and DicerKD + WT exosomes groups at this time point. At day 14, DicerKD hMSCs complemented with WT exosomes had significantly higher (*P* < 0.01) ALP activity compared to WT hMSCs. Complementation of AGO2KD hMSCs with WT exosomes did not significantly change ALP activity at day 7 and 14 (Fig. 3d) and the ALP activity of these cells remained unchanged with respect to the AGO2KD group that did not receive exosomes indicating that the exosomes were ineffective in AGO2KD cells. The insert in Fig. 3d illustrates this more clearly. The addition of either the miR-183-5p or miR-411-5p mimics significantly (*P* < 0.01) improved ALP activity in DicerKD cells after 7 and 14 days. This was more prominent with miR-411-5p mimic (Fig. 3d).

### Osteoinductive gene expression

**Figure 4:**
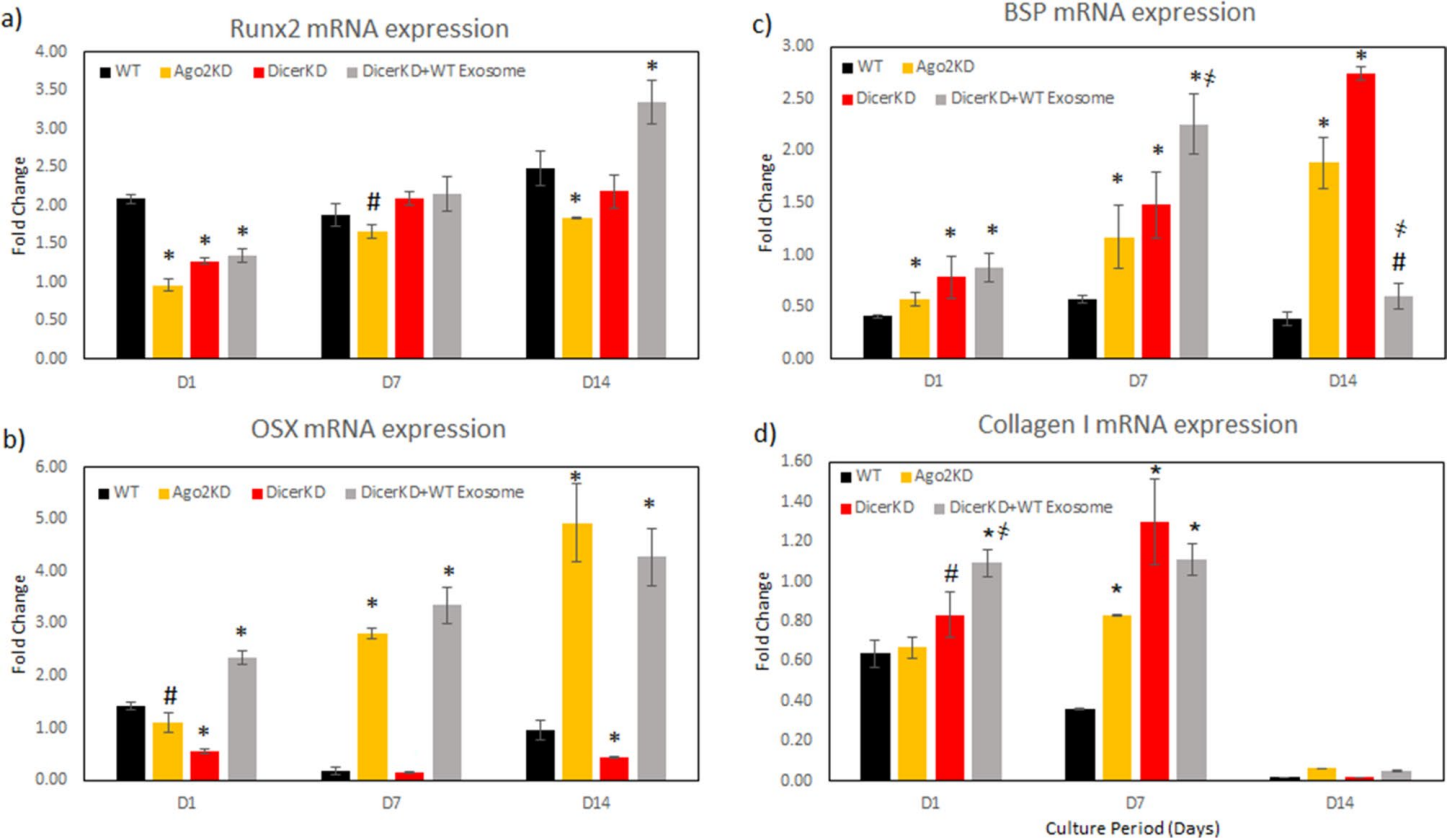
Quantitative gene expression. Expression of osteoinductive genes in WT, DicerKD and AGO2KD hMSCs under differentiation conditions. * denotes to *P* < 0.01 and # denotes to *P* < 0.05 in comparison to WT hMSCs. ҂ denotes significant difference (*P* < 0.01) compared to DicerKD hMSCs. Fold changes are calculated relative to undifferentiated WT hMSCs at day 0. One-way ANOVA and Tukey's post-hoc test was used for all statistical analyses.

The expression pattern of osteoinductive marker genes Runx2, Osterix (OSX), bone sialoprotein (BSP) and type I collagen was evaluated in WT, DicerKD and AGO2KD hMSCs in the presence of osteogenic differentiation medium in vitro. Results indicated that Runx2 mRNA expression was significantly lower (*P* < 0.01) in DicerKD and AGO2KD cells compared to WT hMSCs at day 1 in osteogenic medium. At day 7, Runx2 mRNA expression was elevated in DicerKD and DicerKD + WT exosomes, but not statistically significant. Complementation of DicerKD hMSCs with WT exosomes increased Runx2 mRNA expression at day 14 compared to WT hMSCs (*P* < 0.01) (Fig. 4a). Runx2 expression was significantly lower (*P* < 0.05) in AGO2KD cells compared to WT hMSCs.

OSX mRNA expression was significantly lower in DicerKD (*P* < 0.01) and AGO2KD (*P* < 0.05) compared to WT hMSCs at day 1 in osteogenic medium. Complementation of DicerKD hMSCs with WT exosomes significantly increased (*P* < 0.01) OSX expression at day 1. At day 7, OSX mRNA expression decreased in both WT hMSCs and DicerKD cells (*P* < 0.01). The complementation of DicerKD cells with WT exosomes increased OSX mRNA expression (*P* < 0.001) compared to WT hMSCs (Fig. 4b). These results corroborate well with the mineralization experiments described in the previous section that evaluated calcium deposition and ALP activity.

The effects of AGO2 and Dicer knockdown on BSP mRNA expression appears complex. BSP was significantly higher (*P* < 0.01) in all knockdown groups compared to WT hMSCs at day 1 in osteogenic medium. At day 7 and 14, its expression increased in all groups and its expression was still significantly higher (*P* < 0.01) in AGO2KD, DicerKD and DicerKD + WT exosomes groups. Addition of WT exosomes to DicerKD significantly increased (*P* = 0.01) BSP expression compared to DicerKD hMSCs at day 7. At day 14, DicerKD + WT exosomes group had significantly lower BSP expression than DicerKD hMSCs (*P* < 0.01). However, its expression was still higher (*P* < 0.05) in DicerKD compared to WT hMSCs (Fig. 4c).

Collagen I mRNA expression was increased in all cell types during 1–7 days in culture. When compared to WT hMSCs, Collagen I mRNA expression was increased at day 1 in osteogenic medium in DicerKD and DicerKD + WT exosomes groups (*P* < 0.01). By day 7, both AGO2KD and DicerKD cultures demonstrated increased Collagen I mRNA expression, while it was decreased in WT hMSCs (*P* < 0.01). DicerKD + WT exosomes group had significantly higher (*P* < 0.01) Collagen I mRNA expression compared to WT hMSCs. At day 14, Collagen I mRNA expression was reduced in all groups (*P* < 0.01) (Fig. 4d).

### SMAD 1/5/8 phosphorylation

**Figure 5:**
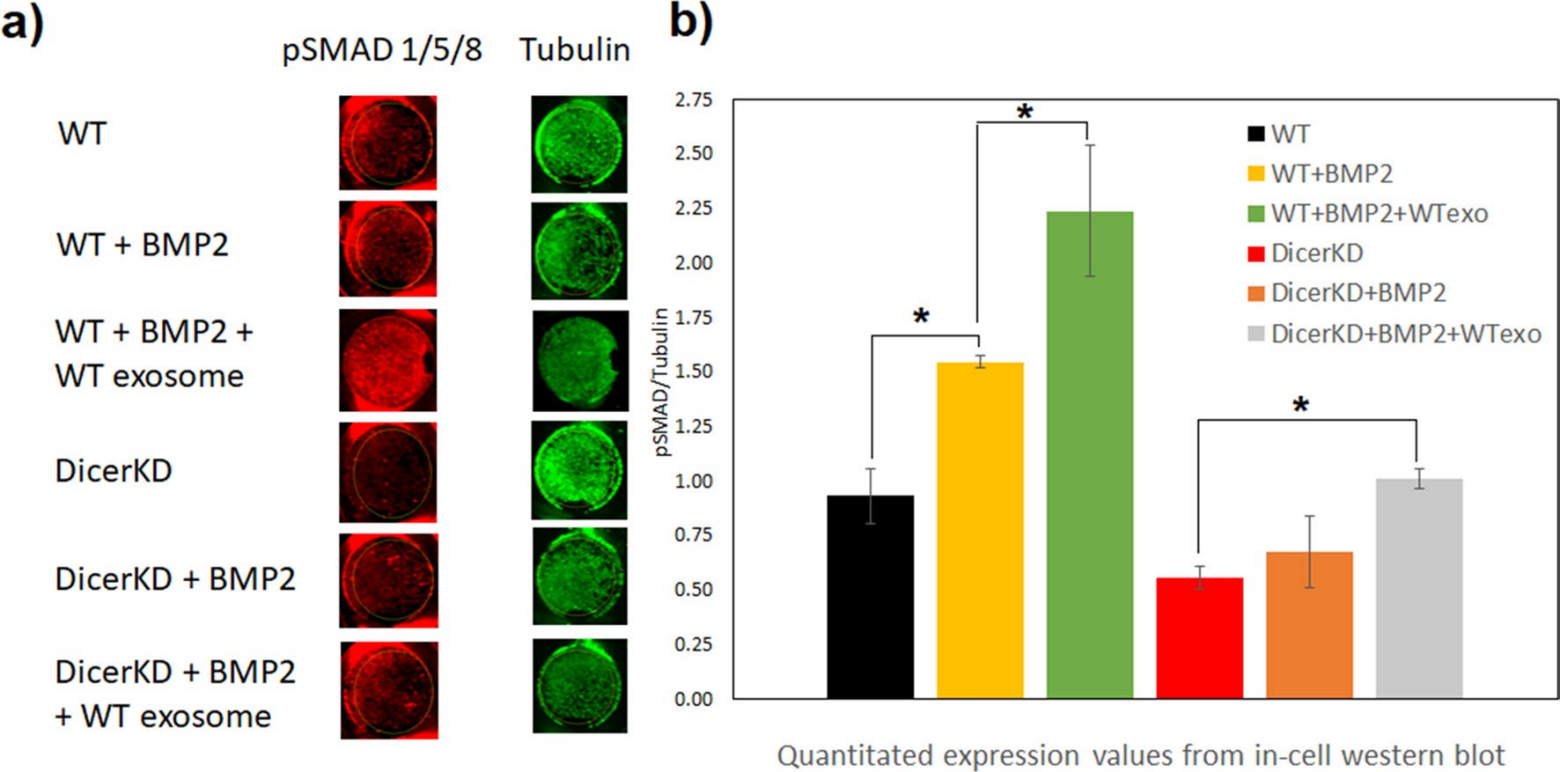
SMAD1/5/8 Phosphorylation in response to rhBMP2 treatment. (**a**) Representative images of individual wells showing phosphorylated SMAD1/5/8 staining (red) and corresponding tubulin expression (green) (n = 3 per each group). (**b**) Quantitation of the images revealed that SMAD 1/5/8 phosphorylation was significantly impaired (*P < 0.01) in DicerKD hMSCs in the presence of BMP2 compared to WT hMSCs. Complementation of DicerKD cells with WT exosomes significantly increased (*P < 0.01) the phosphorylation of SMAD 1/5/8 four hours post rhBMP2 treatment. While these levels were statistically insignificant with respect to WT cells, (*P* > 0.05), the mean value remained lower indicating a partial restoration of BMP2 function with WT exosome complementation. One-way ANOVA and Tukey's post-hoc test was used for statistical analyses.

SMAD 1/5/8 activity is an indicator of the BMP2 signaling pathway activation^16–18^. Here we evaluated the ability of WT and DicerKD hMSCs to respond to the exogenous addition of rhBMP2 at the level of SMAD1/5/8 phosphorylation. Figure 5a shows representative images of individual wells showing phosphorylated SMAD1/5/8 staining (red) and corresponding tubulin expression (green). Quantitation of the fluorescence revealed that SMAD 1/5/8 phosphorylation was significantly impaired (*P* < 0.01) in DicerKD hMSCs treated with rhBMP2 compared to WT hMSCs. Complementation of DicerKD cells with WT exosomes significantly increased (*P* < 0.01) the phosphorylation of SMAD 1/5/8 at 4 h following rhBMP2 treatment (Fig. 5b), indicating a partial restoration of BMP2 function with WT exosome complementation.

### Characterization of cellular miRNA cargo

**Figure 6:**
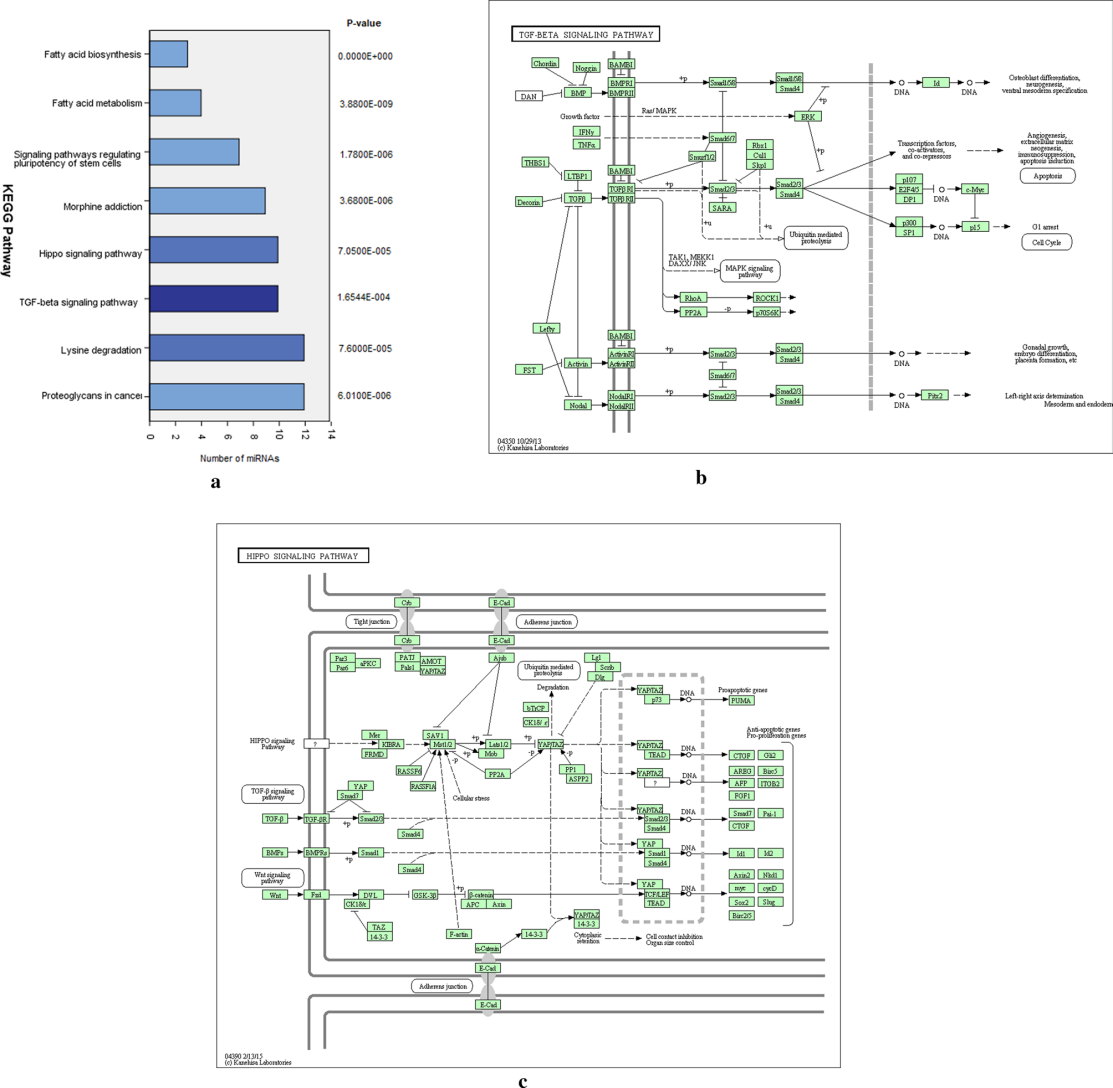
(**a**) KEGG analysis summary. KEGG analysis summary of pathways affected in DicerKD hMSCs. (**b**) TGF-β signalling pathway components identified in the genome and transcriptome WT hMSCs assignment of KEGG orthology terms. microT-CDS predicted miRNAs involved in this pathway include hsa-miR-548w, hsa-miR-6833-3p, hsa-miR-4772-3p, hsa-miR-5683, hsa-miR-548ax, hsa-miR-5000-3p, hsa-miR-548az-5p, hsa-miR-513b-3p, hsa-miR-365b-5p, hsa-miR-708-5p. (**c**) HIPPO signalling pathway components identified in the genome and transcriptome WT hMSCs assignment of KEGG orthology terms. microT-CDS predicted miRNAs involved in this pathway include hsa-miR-3914, hsa-miR-548w, hsa-miR-6833-3p, hsa-miR-5683, hsa-miR-548ax, hsa-miR-5189-5p, hsa-miR-548az-5p, hsa-miR-708-5p, hsa-miR-4738-3p, hsa-miR-320a.

We compared the miRNA cargo of DicerKD and WT hMSCs using miRNA-Seq analysis. The top 50 most abundant miRNAs in WT hMSCs were selected and compared their expression with that of DicerKD hMSCs (Table 1). The results demonstrated that the majority of miRNAs were downregulated in DicerKD cells compared to WT hMSCs. Five miRNAs were upregulated. KEGG pathway analysis was performed to investigate the pathways affected by these 50 miRNAs. We further classified the 25 most down regulated miRNAs in DicerKD cells (Table 2). Multiple pathways were significantly affected by the alteration of miRNA in DicerKD cells (Fig. 6a). Further pathway analysis revealed that the TGFβ and Hippo signaling pathways controlling osteoinduction were targeted by the included miRNAs (Fig. 6b, c).

**Table 1:** Top 50 most abundant miRNAs in WT hMSCs and their expression in DicerKD hMSCs.

Sequence	Number of copies		DicerKD/ WT
	DicerKD hMSCs	WT hMSCs	
hsa-miR-3914	1621	17,162	0.09
hsa-miR-4691-5p	807	16,081	0.05
hsa-miR-6894-3p	45,165	9842	4.59
hsa-miR-548w	1634	7864	0.21
hsa-miR-6501-3p	2113	7106	0.30
hsa-miR-183-5p	886	6965	0.13
hsa-miR-6764-3p	1279	4956	0.26
hsa-miR-320a-3p	6148	4758	1.29
hsa-miR-423-3p	1168	4696	0.25
hsa-miR-3924	650	4575	0.14
hsa-miR-3692-5p	1562	4483	0.35
hsa-miR-6833-3p	1391	3994	0.35
hsa-miR-520 g-5p	1024	3929	0.26
hsa-miR-182-5p	597	3903	0.15
hsa-miR-6889-5p	505	3697	0.14
hsa-miR-6758-5p	2467	3296	0.75
hsa-miR-5092	1358	3207	0.42
hsa-miR-191-5p	26,690	3152	8.47
hsa-miR-8075	663	3076	0.22
hsa-miR-6764-5p	833	2988	0.28
hsa-miR-4772-3p	2152	2911	0.74
hsa-miR-1276	1548	2865	0.54
hsa-miR-103a-3p	18,502	2774	6.67
hsa-miR-4754	840	2708	0.31
hsa-miR-375-3p	1142	2663	0.43
hsa-miR-151a-3p	20,667	2562	8.07
hsa-miR-6756-3p	571	2451	0.23
hsa-miR-5683	361	2410	0.15
hsa-miR-3660	230	2407	0.10
hsa-miR-548ax	879	2381	0.37
hsa-miR-107	1233	2280	0.54
hsa-miR-5000-3p	394	2221	0.18
hsa-miR-6745	709	2208	0.32
hsa-miR-5189-5p	689	2162	0.32
hsa-miR-548az-5p	814	2161	0.38
hsa-miR-513b-3p	610	2137	0.29
hsa-miR-6827-5p	1660	2056	0.81
hsa-miR-4634	2034	2045	0.99
hsa-miR-548au-3p	571	2017	0.28
hsa-miR-4497	584	2012	0.29
hsa-miR-600	203	1975	0.10
hsa-miR-1200	971	1971	0.49
hsa-miR-4706	308	1964	0.16
hsa-miR-3131	446	1955	0.23
hsa-miR-365b-5p	243	1950	0.12
hsa-miR-19a-3p	1542	1937	0.80
hsa-miR-515-5p	29,150	1909	15.27
hsa-miR-455-5p	650	1907	0.34
hsa-miR-708-5p	2677	1877	1.43
hsa-miR-4738-3p	669	1876	0.36

**Table 2:** Top 25 downregulated miRNAs in DicerKD hMSCs compared to WT hMSCs.

Sequence	Number of copies		DicerKD/WT
	DicerKD hMSCs	WT hMSCs	
miR-6830-3p	0	33	0.00
miR-320e	0	19	0.00
miR-4739	13	512	0.03
miR-4691-5p	807	16,081	0.05
miR-12114	7	119	0.06
miR-1207-3p	7	70	0.09
miR-3914	1621	17,162	0.09
miR-3660	230	2407	0.10
miR-6864-3p	72	720	0.10
miR-600	203	1975	0.10
miR-1321	39	373	0.11
miR-411-5p	144	1262	0.11
miR-6886-5p	20	162	0.12
miR-365b-5p	243	1950	0.12
miR-3125	112	889	0.13
miR-6820-5p	66	520	0.13
miR-183-5p	886	6965	0.13
miR-6889-5p	505	3697	0.14
miR-6786-5p	98	702	0.14
miR-3924	650	4575	0.14
miR-7109-5p	184	1276	0.14
miR-518b	20	136	0.14
miR-7847-3p	223	1544	0.14
miR-5683	361	2410	0.15
miR-182-5p	597	3903	0.15

## Discussion

MSCs are multipotent cells with defined ability to differentiate to bone, cartilage, and adipose tissues^19^. Osteoblastic differentiation of MSCs can be achieved in cell culture by morphgen induction using BMPs^20^, by media supplementation^21^, and by nanotopographic cues^22^. This experimental experience with MSCs has fostered great enthusiasm for the use of MSCs for clinical bone regeneration^23^. MSCs have been used as the cellular foundation for tissue engineered bone substitutes based on diverse combinations of scaffold materials and cytokines or growth factors^24^. However, there remain many challenges in translating MSCs to clinical practice of bone regeneration.

The function of MSCs in regeneration may involve direct biosynthetic roles or indirect roles for instructing targeted tissues and cells in regenerative processes. The possible paracrine actions of MSCs in tissue regeneration implies that biomolecules produced by the MSC elicit tissue repair or regeneration^25^. The implantation of MSCs in effort to promote tissue regeneration has been shown to modulate the immune response, induce progenitor cell differentiation, suppress apoptosis, and enhance autophagy^26^. These effects are associated with the MSCs secretion of factors critical for instruction of local tissue responses^27^. The MSC secretome is composed of soluble proteins, nucleic acids, lipids and extracellular vesicles. MSC conditioned media can substitute for the MSC in directing bone regeneration^28^. More recent studies have extended our understanding of exosomes as key signaling components of the MSC secretome^5,29,30^ and the treatment of experimental osseous defects with MSC exosomes enhances bone regeneration^31–33^. This use of MSC exosomes in bone regeneration has potential advantages versus MSCs themselves. Included are the avoidance of concerns of immunogenicity and tumorigenicity, potential storage advantages as off-the-shelf material (for use in acute scenarios), and the large-scale production from selected or engineered cell lines to provide specifically engineered exosomes to direct specific cellular functions. These functions are, in part, the result of miRNA actions in target cells.

Among the exosome cargo, miRNAs are known to positively influence osteoinduction and bone regeneration^34^. However, there remains controversy regarding the significance of the miRNA cargo in signaling of osteogenesis. The protein cargo is also implicated in MSC exosome effects^35^. In the present study, we demonstrate that alteration of the miRNA cargo by knockdown of Dicer impairs hMSC differentiation. This was evident at the level of osteoinductive gene expression and culture mineralization and is consistent with a previous report implicating Dicer expression in bone regeneration^36^. Here, the reduction of otherwise highly expressed miRNAs that resulted from the knockdown of Dicer in hMSCs was associated with the reduced differentiation of DicerKD hMSCs when compared to WT hMSCs at the level of ALP activity, osteogenic gene expression and mineralization. Under mineralizing conditions in vitro, the reduction in miRNAs following knockdown of Dicer expression altered the progression of osteogenic differentiation.

Complementation experiments conducted by adding WT exosomes to DicerKD hMSC cultures resulted in the recovery of osteoinductive gene expression and culture mineralization, suggesting that the exosomes produced by WT MSC cultures contain osteoinductive cargo. With respect to ALP activity, complementation with WT exosomes was able to rescue the deficiency in DicerKD hMSCs but not in AGO2KD hMSCs indicating that the exosomal effects may primarily be mediated by exosomal cargo. In an effort to understand if transfer of miRNA via exosomes can be quantified, we evaluated the miRNA levels of some miRNAs that are prominent in MSC exosomes in WT and DicerKD cells treated with WT exosomes and compared them to untreated cells (supplementary Fig. 4). The results clearly showed that the miRNA transfer from exosomes to cells can be quantified and as expected, and the increase in miRNA levels was more apparent in the DicerKD cells compared to WT cells due the reduced levels of miRNA presence. Our miRNA-Seq and pathway analyses show that Dicer knockdown reduced miRNA expression that affected pathways central to hMSC differentiation including TGF-β pathway, hippo pathway and ECM biosynthesis pathways. Many studies indicate that individual miRNAs contribute to the regulation of osteoinduction and osteogenesis^37,38^. In the present study, the treatment of DicerKD cells with two miRNA mimics that were significantly downregulated in DicerKD cells (mir183-5p and miR411-5p) partially recovered cultured DicerKD cell osteoinduction. Based on miRNA-Seq data, we selected two miRNA (mir183-5p and miR411-5p) that demonstrated high levels of expression in WT hMSCs and marked reduction in Dicer KD cells. While other miRNAs with significant fold reduction could have been selected, their expression in WT hMSCs was relatively low. We did not select uncharacterized miRNAs for this purpose. These results highlight the possibility of complementing lost function or correcting dysfunction using miRNA mimics or the exosomes of hMSCs.

The relative abundance of six miRNAs were actually increased in DicerKD hMSCs. Included were miR-515-5p, miR151a-3p, miR-103a-3p, miR191-5p, miR320a-3p, and miR6894-3p. Increased expression of miRNAs with reduced Dicer activity is not unexpected as miRNAs may act at selected gene loci to repress mRNA/miRNA expression at these loci. miR 320a-3p is implicated in the inhibition of osteoblast differentiation via HOXA10^39^. miR 515-5p was a downregulated miRNA in osteoblasts compared to undifferentiated unrestricted somatic stem cells^40^. Interestingly miR 515-5p was upregulated during chondrogenesis^41^. miR103a-3p is known to inhibit DKK1 expression^42^ to affect osteogenesis and is found in multiple myeloma extracellular vesicles that may inhibit osteogenesis^43^. miRNA-191-5p is considered an endogenous miRNA in hMSCs^44^, but in DicerKD cells it is 8.47 fold higher than in WT hMSCs. These miRNAs may play a role in the complex gene regulation observed at the level of osteoinductive mRNA expression observed in Fig. 4.

Osterix expression was markedly reduced throughout the osteoinductive culture of DicerKD cells. It may be a key target of miRNA-mediated reduction in osteogenesis. Multiple miRNAs target Osterix gene expression and reduce Osterix levels and function in differentiating osteoblastic cells. Included are miR-96^45^, miR-214^46^, miR-143^47^ and miR145^48^ among others. miR-322 indirectly modulates Osterix protein expression by targeting ToB2^49^. It is interesting that none of these miRNAs were among the 50 most prominent species in hMSC exosomes, suggesting that the miRNA control of Osterix mRNA expression is under marked direct or indirect miRNA control^50^. The miRNA mediated regulation of Osterix mRNA expression and related osteogenesis in cultured hMSCs merits further investigation.

In the present study, the reduction in AGO2 expression led to impaired osteogenesis as observed at the level of ALP expression, culture mineralization and early osteoinductive gene expression. AGO2 is integral to miRNA RISC complex formation and is critical to miRNA directed mRNA degradation^11,12^. The known function of AGO2 in human cells during miRNA mediated gene regulation strongly implicates miRNAs in the regulation of hMSC osteogenesis in culture. AGO2 is a component of the RISC complex that functions at perfect or near-perfect miRNA pairing with target genes to mediate mRNA cleavage. It is the only member of the Argonaute family that possesses endonuclease activity to regulate miRNA guided gene silencing^51^. AGO2 may also increase miRNA stability, but beyond this it is not known to have other functions beyond its interaction with miRNAs and targeted mRNAs. The present knockdown of AGO2 likely has generalized miRNA-mediated effects on osteoinduction and osteogenesis that recapitulate similar findings from other systems such as monocyte differentiation and angiogenesis^52,53^. In previous work using AGO2KD hMSCs, engineered exosomes failed to exert the effect of overexpressed miRNAs^32^, again indicating the significance of AGO2 in miRNA function within target cells. In this study, we have used the AGO2KD cells to implicate a role for exosomal miRNA. While ALP activity was rescued in DicerKD cells, AGO2KD cells displayed no change in ALP activity upon complementation with WT exosomes under mineralizing conditions indicating that AGO2 is required for the exosomal effects to be realized and indirectly indicaing the importance of exosomal miRNA to exosome function.

Overall, the results of these experiments further implicate the role of exosome miRNAs in hMSC osteogenic differentiation. The results also show that it is possible to restore miRNA mediated function in DicerKD cells by complementation with exosomes derived from WT hMHCs. Apart from highlighting the effects of the exosomal miRNA cargo, these results also show the impact of exosomal miRNAs on the osteogenic differentiation of hMSCs. Further investigation at the level of individual or families of miRNAs offer direct opportunities for deploying exosomes and miRNAs to achieve specific regenerative functions.

## Materials and methods

### Generation of Dicer and Argonaute 2 knocked-down hMSCs

Human bone marrow primary mesenchymal stem cells (hMSCs) were purchased from Lonza. HMSCs were transduced with Dicer and Argonaute 2 short hairpin RNA (shRNA) encoding lentiviral particles (Santa Cruz, sc-40489-V and sc-44409-V) according to the manufacturer’s protocol. Transduced cells were stably selected using Puromycin and knockdown was verified by immunoblotting for the respective proteins and RT-qPCR.

### Dicer and Argonaute 2 gene expression knock-down monitoring

Dicer mRNA levels in Dicer knock-down (DicerKD) and wild type (WT) hMSCs, and Argonaute 2 mRNA levels in Argonaute2 knock-down (AGO2KD) and WT hMSCs were assessed using RT‑qPCR at day 1, 7 and 14 in osteogenic medium (αMEM growth medium containing 100 µg/ml ascorbic acid, 10 mM β-glycerophosphate, and 10 mM dexamethasone) (Primers are listed in Table 3). Dicer and Argonaute 2 protein levels were tested in all 3 cell types at day 1 using immunoblotting (Abcam, ab186733, 1/1000 for Argonaute 2; ab14601, 1/250 for Dicer; and Sigma, T5168, 1/2000 for Tubulin).

**Table 3:** Primer sequences used for RT-qPCR.

Gene	Forward (5′ → 3′)	Reverse (5′ → 3′)	Amplicon size (bb)
GAPDH	CAGGGCTGCTTTTAACTCTGG	TGGGTGGAATCATATTGGAACA	102
DICER	GAGCTGTCCTATCAGATCAGGG	ACTTGTTGAGCAACCTGGTTT	92
ARGOUNATE 2	TCCACCTAGACCCGACTTTGG	GTGTTCCACGATTTCCCTGTT	157
RUNX2	TGGTTACTGTCATGGCGGGTA	TCTCAGATCGTTGAACCTTGCTA	101
OSX (Sp7)	CCTCTGCGGGACTCAACAAC	AGCCCATTAGTGCTTGTAAAGG	122
BSP (SPP1)	GAAGTTTCGCAGACCTGACAT	GTATGCACCATTCAACTCCTCG	91
Collagen I	GAGGGCCAAGACGAAGACATC	CAGATCACGTCATCGCACAAC	140

### Proliferation assays

In order to assess the effect of miRNAs on hMSC proliferation, WT, DicerKD and AGO2KD hMSCs were seeded in four 96-well plates (Day 0, each plate containing 0.01 × 10^4^ cells/well, 8 wells per each cell type) in growth medium (αMEM containing 20% fetal bovine serum, 1% l-Glutamine, and 1% antibiotic–antimycotic solution). The next day (Day 1), medium was changed to either regular or osteogenic (100 µg/ml ascorbic acid, 10 mM β-glycerophosphate, and 10 mM dexamethasone) in quadruplicate per cell type. Osteoblastic proliferation was assessed at 1, 3, 5 and 7 days using a colorimetric method (CellTiter 96 Aqueous One Solution, Promega, G3582) according to the manufacturer’s protocol. The absorbance was recorded at 490 nm using a 96-well plate reader (Biotek).

In order to implicate specific miRNAs affected by knockdown of Dicer or AGO2, the effect of miR-183-5p and miR-411-5p mimics (Dharmacon, C-300559–07, C-300987-01) on the proliferation of DicerKD hMSCs was examined. Cells were seeded in four 96-well plates (each plate containing 1 × 10^4^ cells/well, 4 wells/group) in regular growth medium. The next day (Day 0), DicerKD cells were transfected with 25 nM final concentration of either miR-183-5p or miR-411-5p mimics using Lipofectamine3000 according to the manufacturer’s protocol. Transfection was repeated on Day 3. Osteoblastic proliferation was assessed over a 7-day period at day 1, 3, 5 and 7 as described above.

### Osteogenic differentiation and complementation assays

**Figure 7:**
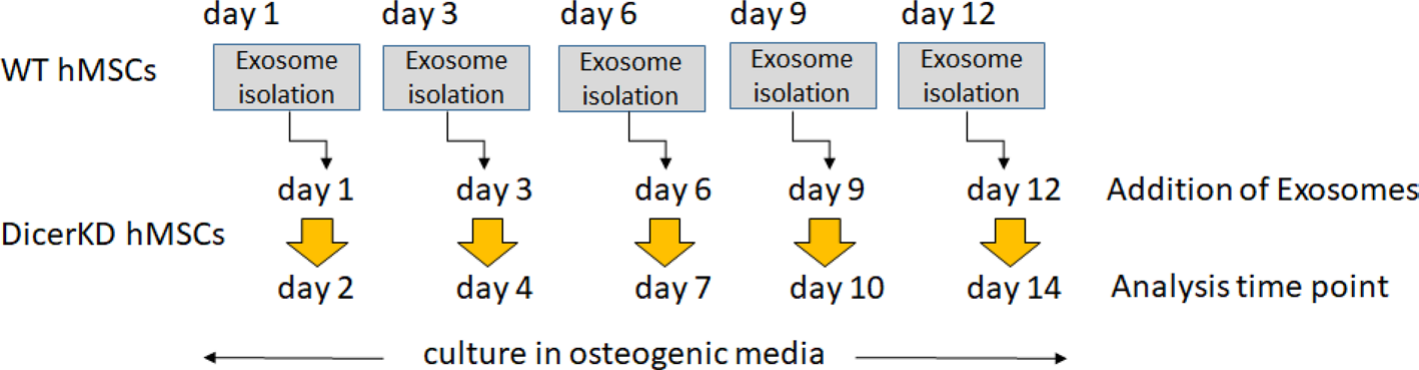
Schematic of experimental design for exosomal complementation study: WT hMSC cultures were initiated 24 h prior to DicerKD and AGO2KD cultures. At designated times, WT hMSCs were grown 24 h in serum free media before collection of media for isolation of WT hMSC exosomes. The following day, WT hMSC exosomes were added to DicerKD and AGO2KD cultures. The cells were harvested at day 1 and after 7 or 14 days for analysis of osteoblastic gene expression and culture mineralization.

WT, DicerKD and AGO2KD hMSCs were seeded in quadruplicate in multiple 6-well plates (0.1 × 10^6^ cells/well) in growth medium. The next day (Day 1), growth medium was replaced with osteogenic medium and cells were cultured for 14 days to induce osteoblast differentiation. In order to implicate the role of Dicer and miRNAs in the process of osteoblastic differentiation, a complementation assay was also performed using WT hMSC-derived exosomes to deliver miRNAs of WT hMSCs to DicerKD and AGO2KD cells undergoing osteoblastic differentiation. For this purpose, a back-to-back cell culture design was implemented. In brief, sets of WT cells were cultured in osteogenic medium (the same passage number and grown under similar culture condition) 2 days prior to the start of main osteogenic differentiation experiment to harvest exosomes. Each plate was used once to collect exosomes. This allowed for the addition of time point-specific (differentiation-specific) exosomes to the test cell cultures. Therefore, exosomes from equal numbers of WT hMSCs were harvested at day 1, 3, 7 and 10, and were added to the DicerKD and AGO2KD cell cultures at day 1, 3, 7 and 10. The final configuration of osteogenic cell culture was as follows: WT, DicerKD, DicerKD + WT exosomes, AGO2KD and AGO2KD + WT exosomes (Fig. 7).

### Exosome isolation

For exosome isolation, WT hMSCs in osteogenic medium were washed with PBS, and then serum free osteogenic medium was added. After 24 h, the serum free media was harvested and centrifuged for 15 min at 1500×*g* to remove cell debris, and then was concentrated 5 times using a 100 KDa spin filter (Millipore). ExoQuick TC isolation reagent (System Biosciences) was used to isolate exosomes overnight according to the manufacturer's protocol.

### Alizarin Red staining

Calcium deposition and mineralization was assessed using Alizarin Red staining in quadruplicate for each of WT, WT + WT exosomes, DicerKD, DicerKD + WT exosomes, and AGO2KD hMSCs groups at days 1, 7 and 14. Cells were washed with PBS and fixed with 4% neutral buffered PFA (pH 7.2) and kept in 4 °C until staining. All culture plates were stained at the same time with 2% Alizarin Red solution (pH 4.1) for 5 min at room temperature. The plates were washed twice with ddH2O and air dried before scanning.

Quantitation of Alizarin Red staining was performed spectrophotometrically. The stained wells were bleached using 1 ml of 10% acetic acid and 20% methanol solution for 15 min. The liquid absorbance of 100 µl of collected liquid was measured at 405 nm. Absorbance was normalized to cell number by counting the number of nuclei per well following permeabilization and staining with DAPI (VECTASHIELD, H-1200-10). Nuclei were visualized using a BioRad fluorescent microscope (4 fields per well) and the number of nuclei was digitally counted using EVOS FL Auto software (Version 31201). The absorbance reading from each well was then divided by the average number of nuclei per well to standardize the reading among different samples.

### Alkaline phosphatase (ALP) activity assay

ALP activity was measured at days 1, 7 and 14 of osteoinduction in the WT, DicerKD, DicerKD + WT exosomes, AGO2KD and AGO2KD + WT exosomes hMSCs groups in quadruplicate using a colorimetric assay kit (Abcam, ab83369). At each time point, cells were washed with PBS and collected with assay buffer and kept on ice for further processing. The assay procedure was performed according to the manufacturer's protocol using 45 µg protein from each sample. Output was measured at OD 405 nm on a microplate reader (Biotek). Calculations were performed following the product’s protocol.

In addition, the effect of miR-183-5p and miR-411-5p mimics (Dharmacon, C-300559-07, C-300987-01) on the differentiation of DicerKD hMSCs was investigated. DicerKD cells were seeded in two 12-well plates (each plate containing 10^4^ cells/well, 4 wells per each group) in regular growth medium. The next day (Day 0), DicerKD cells were transfected with 25 nM final concentration of either miR-183-5p or miR-411-5p mimics using Lipofectamine3000 according to the product’s protocol. Transfection was repeated every 3 days. ALP activity was measured at days 1, 7 and 14 in each group as described above.

### RNA isolation and RT‑qPCR

RNA was extracted from WT, DicerKD, DicerKD + WT exosomes, and AGO2KD hMSCs groups at days 1, 7 and 14 following osteoinduction using miRNeasy Mini kit according to the manufacturer’s protocol. 1 μg total RNA was used for cDNA synthesis using RevertAid RT kit (Thermoscientific, Ref. K1691) according to the manufacturer's protocol, and RT‑qPCR was performed using SYBR Green PCR reagent (applied biosystems) with primers listed in Table 3. Expression levels were normalized against GAPDH and relative expression levels and fold changes of each gene were calculated via the 2^−ΔΔCT^ method.

In order to demonstrate the successful delivery of miRNAs to DicerKD and WT hMSCs by means of WT exosomes, we isolated WT exosomes from 32 ml serum free media, resuspended the exosomes in 1600 µl of PBS and added to DicerKD and WT hMSCs cultured in 6 well plate in quadruplicate (200 µl of exosomes/well). After 2 h, the cells were harvested from DicerKD, DicerKD + WT exosomes, WT and WT + WT exosomes hMSCs groups and miRNA was isolated using miRNeasy Mini kit according to the manufacturer's protocol. 2 µg of total RNA was used to generate cDNA using miScript II RT Kit (Qiagen, Cat No. 218161) according to the manufacturer's protocol. RT‑qPCR was performed using miScript SYBR Green PCR kit (Qiagen, 1046470) according to the manufacturer's protocol with primers listed in Table S1. The selected miRNAs were highly abundant in WT exosomes and were downregulated in DicerKD cells according to our sequencing data. Expression levels were normalized against hsa-RNU-6B and relative expression levels and fold changes of each miRNA in WT + WT exosomes and DicerKD + WT exosomes groups were calculated via the 2^−ΔΔCT^ method relative to WT and DicerKD group, respectively.

### miRNA sequencing analysis

Libraries were constructed using 500 ng of total RNA from WT, DicerKD, and AGO2KD using TruSeq Small RNA Sample Prep Kit (Illumina). Libraries were multiplexed and sequenced on a HiSeq 2500 using TruSeq Rapid SBS sequencing chemistry v2. Fastq files were generated with the bclfastq v1.88.4 and adapter sequences and low-quality sequences were removed and miRNAs were identified with miRbase. To explore possible osteoinductive/osteogenic pathways impacted by miRNAs differentially expressed in DicerKD hMSCs, KEGG analysis of top 50 abundant miRNAs were performed using DIANA-miRPath v3. The heatmap of miRNAs was generated based on pathways union.

### Assessment of SMAD 1/5/8 phosphorylation

DicerKD and WT hMSCs were seeded in 96-well plate (0.01 × 10^6^ cells/well; 6 groups in total, 3 triplicates of DicerKD and 3 triplicates of WT hMSCs) in regular media. The next day, 250 ng/ml rhBMP2 was added to one DicerKD and one WT triplicate group. In one other WT and DicerKD group, 250 ng/ml of rhBMP2 and 20 μl/well of WT exosomes were added to the culture. WT exosomes were isolated prior to the start of experiment from 4 ml of serum free media and resuspended in 200 μl of regular media. Four hours after treatment, the cells were washed with PBS and fixed with 4% neutral buffered PFA, permeablized and immunostained for phosphorylated SMAD 1/5/8 and tubulin with the corresponding primary and secondary antibodies. The plates were scanned using a Licor Odyssey CLX imager. The fluorescence within the wells was quantitated using the image analysis software (Image Studio) provided with the instrument. The readings were normalized to tubulin expression.

### Statistical analysis

The normal distribution of the data obtained from the experiments was evaluated using the Shapiro–Wilk test. For experiments involving two groups, Mann–Whitney U test with a confidence interval of 95% was utilized. For the experiments involving comparison of more than two groups, one-way ANOVA was performed with a confidence interval of 95%. Pairwise comparisons were performed using Tukey’s ad-hoc test with a confidence interval of 95%.

## Supplementary Information

Supplementary Informations.
