# Supplementary material for: The importance of cellular and exosomal miRNAs in mesenchymal stem cell osteoblastic differentiation
Source: Sci Rep. 2021 Mar 15;11:5953. doi: 10.1038/s41598-021-85306-2 (PMC7960990; doi:10.1038/s41598-021-85306-2)
Supplement: Supplementary file 1 — Supplementary Informations. [file 41598_2021_85306_MOESM1_ESM.docx]

**Supplementary Figures:**

**The Importance of Cellular and Exosomal miRNAs in Mesenchymal Stem Cell Osteoblastic Differentiation**

*Sajjad Shirazi, Chun-Chieh Huang, Miya Kang, Yu Lu, Sriram Ravindran^*^ and Lyndon F Cooper^*^*

*From the Department of Oral Biology, College of Dentistry, University of Illinois at Chicago, Chicago, Illinois 60612*

**Address correspondence to (*):**

Lyndon F Cooper

Department of Oral Biology

University of Illinois College of Dentistry

801 S Paulina St., Room 561C

Chicago, Illinois 60612

cooperlf@uic.edu

Sriram Ravindran

Department of Oral Biology

University of Illinois College of Dentistry

801 S Paulina St., Room 561C

Chicago, Illinois 60612

sravin1@uic.edu

**Running Title**: miRNAs in MSC Differentiation.

**
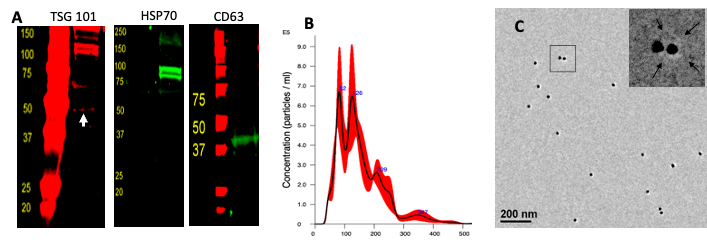
Supplementary Figure 1:**

**Supplementary Figure** 1: **Characterization of MSC derived exosomes.**

Immunoblots of exosomal lysates showing the presence of TSG 101 (white arrow), HSP70 and CD63 markers. B) NTA analysis of the isolated vesicles showing its size distribution. C) TEM image of immunogold labelled exosomes showing the presence of CD63. The insert is a magnified image of the boxed region. The arrow in the insert point to the exosomal membrane.

*NOTE: The three included images in panel A represent digitally captured fluorescence (LICOR) images of proteins transferred to membranes that were cut prior to probing. These figures represent our source data and are complete in their representation without modification or cropping.*

**Supplementary figure 2:**

**Effect of Ago2 and Dicer knockdown on hMSC Adpiogenic Differentiation**.

WT, DicerKD and Ago2KD hMSCs were cultured for 2 weeks in Adipogenic media (alpha-MEM + 20% FBS + 1% Anti-Anti + 1 μM dexamethasone+ 500 μM 3-Isobutyl-1-methylxanthine (IBMX)+ 100 μM indomethacin+ 10 μg/ml insulin). **a)** The cells were stained using Oil Red O staining for Lipid droplets after 2 weeks. The results demonstrated considerable difference between WT, DicerKD and Ago2KD hMSCs. **b)** RNA was extracted at Day 0, 7 and 14 to assess the expression of adipogenic genes in different cell types. * Denotes to P<.001 as tested with one-way ANOVA and post-hoc Tukey test. Fold changes are calculated compared to WT D1 in regular media.

Ref: Adipogenic Differentiation of hMSCs is Mediated by Recruitment of IGF-1r Onto the Primary Cilium Associated With Cilia Elongation. Melis D et al. Stem Cells, May 21, 2015

**Supplemental Figure 3:**

Full length blots for **Figure 2c. Characterization of Dicer and AGO2 knockdown**:

Figure 2 c)Dicer Figure 2c) Argonaute 2


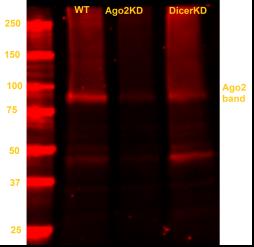

Figure 2c) Tubulin


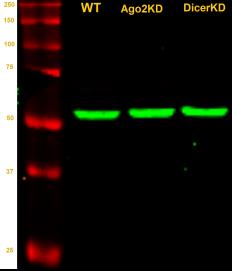


*NOTE: These three images represent digitally captured fluorescence (LICOR) images of proteins transferred to membranes that were cut prior to probing. These figures represent our source data and are complete in their representation of the probed membranes without modification or cropping.*

Supplemental table 1. Primer sequences used for miRNA RT-qPCR

| **miRNA** | **Sequence 5’ to 3’** |
| --- | --- |
| hsa-let-7i-5p | GGGTGAGGTAGTAGTTTGTGCTGTT |
| hsa-miR-199a-3p | GGGACAGTAGTCTGCACATTGGTTA |
| hsa-let-7a-5p | GGGGTGAGGTAGTAGGTTGTATAGTT |
| hsa-miR-16-5p | GGTAGCAGCACGTAAATATTGGCG |
| hsa-miR-221-3p | GAGCTACATTGTCTGCTGGGTTTC |
| hsa-RNU-6B | GCCCCTGCGCAAGGATGAC |

**Supplementary Figure 4:**


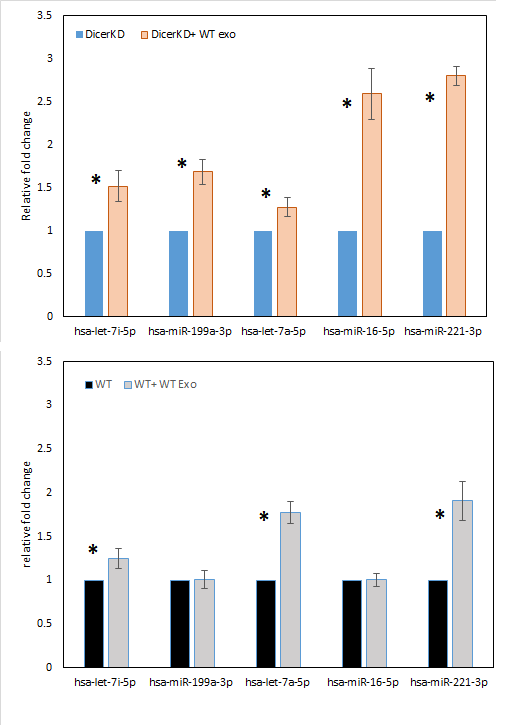


In order to demonstrate the successful delivery of miRNAs to DicerKD hMSCs by means of WT exosomes, WT and DicerKD hMSCs cultured in 6 well plates in quadruplicate were incubated with WT exosomes. After 2 hours, miRNA was isolated from WT and DicerKD cells +/- exosomes and RT‑qPCR was performed. The expression levels of selected miRNAs were normalized against hsa-RNU-6B and relative expression levels and fold changes of each miRNA in WT+ WT exosomes and DicerKD+WT exosomes groups were calculated via the 2^‑ΔΔCT^ method relative to respective untreated controls. * denotes statistically significant difference (P<0.01) measured by student’s t-test
